# Rapid Degradation of Nitrochlorobenzene by Activated Persulfate Oxidation With Biochar Supported Nanoscaled Zero Valent Iron

**DOI:** 10.3389/fchem.2021.615694

**Published:** 2021-03-10

**Authors:** Xiang Wang, Rufeng Deng, Wenbo Shen, Jianbo Huang, Qun Li, Yuanchao Zhao, Jinzhong Wan, Yan Zhou, Tao Long, Shengtian Zhang

**Affiliations:** ^1^ State Environmental Protection Key Laboratory of Soil Environmental Management and Pollution Control, Nanjing Institute of Environmental Sciences, Ministry of Ecology and Environment, Nanjing, China; ^2^ College of Environment, Hohai University, Nanjing, China

**Keywords:** persulfate, biochar, zero valent iron, nitrochlorobenzen, kinetics

## Abstract

Although pesticide intermediates are a kind of typical toxic pollutant in contaminated sites, the remediation of these contaminants in groundwater and soils is of limited concern. In the present study we investigated the performance of a novel heterogeneous oxidation system, biochar supported nanoscaled-zero valent iron (nZVI/BC) activated persulfate (PS), in the oxidative degradation of nitrochlorobenzene (NCB), a typical pesticide intermediate. Peanut shell based nZVI/BC was prepared and used as the PS activator. The degradation kinetics of m-, p-, and o-NCB isomers in the aqueous phase were investigated. The effects of BC/nZVI composition (Fe/BC mass ratio), the amount of BC/nZVI and PS, and initial contaminant concentration on NCB removal were also examined. Results suggest that over 90% removals of three NCB isomers could be obtained by the nZVI/BC activated PS system at initial NCB concentration of 10 mg L^−1^. The combination of nZVI/BC composite and PS showed superior performance to PS alone. The optimal treatment condition was supposed as the Fe: BC ratio of 1:1, Fe amount of 6 mmol L^−1^, and the mole ratio of Fe to perfulfate of 1:1.

## 1 Introduction

Nitrochlorobenzene (NCB) is a kind of chlorine-containing nitro aromatic hydrocarbon compound, which is widely used as a basic chemical in dyeing, explosives, pesticides, and chemical synthesis industries (Li and Zhu, 2014; Wu et al., 2014; Vahid et al., 2015; Zhang et al., 2019). The world's annual discharge of NCB compounds into the environment is about 30, 000 tons. China is one of the countries with the largest production of ortho and para-nitrochlorobenzene in the world (Chen et al., 2018). NCB has strong carcinogenic and mutagenicity, a very stable structure, and it is not easily decomposed and transformed in the environment. NCB can enter the human body and animals through inhalation, ingestion, and skin absorption, causing tissue lesions in the lungs, breast, liver, kidneys and other organs, accompanied by clinical symptoms such as mental disorders and coma (Le et al., 2011). Therefore, NCB has been listed as the environmental priority control pollutants by the United States EPA and China SEPA.

Varied physical, chemical, and biological technologies have been explored to treat NCB in water (Le et al., 2011; Li and Zhu, 2014; Huang et al., 2016; Liu et al., 2016; Chen et al., 2018; Song et al., 2020). Among which advanced oxidation technology (AOT) is more reliable due to its high degradation efficacy and lower secondary pollution. Persulfate oxidation, as a new and green AOT, has been increasingly investigated and widely used for *in situ* chemical oxidation (ISCO) (Liu et al., 2015; Devi et al., 2016; Fang et al., 2017; Zhu et al., 2017; Park et al., 2019; Khashij et al., 2020; Lee, et al., 2020). Persulfate is stable, and has a much longer lifetime in the subsurface than hydrogen peroxide. To promote the performance of persulfate, various activation methods are usually applied to activate persulfate to generate reactive radical species, mainly sulfate radicals and hydroxyl radicals, which can oxidize most recalcitrant organic contaminants (Fang et al., 2017; Ghanbari and Moradi, 2017; Zhu et al., 2017). Heat-activation, alkali activation, and transition metals activation are most frequently used.

Very recently a novel activation method, persulfate activated by biochar supported nano zero valent iron (nZVI/BC), was investigated, and exciting results were found in the treatment of chlorinated organics (Yan et al., 2015; Yan et al., 2017; Xu et al., 2018; Hayat et al., 2019; Luo et al., 2019). Yan et al. (2015) synthesized nZVI/BC composite as an activator for persulfate to enhance the removal of trichloroethylene (TCE) in aqueous solutions. Reliable treatment performance was obtained, much higher than that of nZVI/BC or persulfate alone. Similar results were reported for the degradation of nonylphenol, imidacloprid and diatrizoate (Hussain et al., 2017; Xu et al., 2018; Luo et al., 2019). As demonstrated by the following process in Eqs 1–3, the enhanced formation of the SO^−^_4_. was detected and supposed as the main mechanism for the accelerated degradation of pollutants. However, the application of the above promising persulfate activation technique in the treatment of NCB has not been mentioned.

$$Fe^{0}+H_{2}O+0.5O_{2}\to Fe^{2+}+2OH^{-}$$ (1)

(2) $$Fe^{0}+2H_{2}O\to Fe^{2+}+H_{2}+2OH^{-}$$

(3) $$S_{2}O_{8}^{2-}+Fe^{2+}\to Fe^{3+}+SO_{4}^{2-}+\;SO_{4}^{-}.$$

The present study aimed at exploring the feasibility of treating three NCB isomers by nZVI/BC activated PS. Various parameters including the BC-Fe ratio, the dosage of nZVI/BC and PS, as well as the initial NCB concentration were examined. Both degradation percentage and kinetics of NCB were analyzed and compared to obtain the optimal reaction conditions.

## 2 Materials and Methods

### 2.1 Materials

Ferrous sulfate heptahydrate was purchased from Sinopharm Chemical Reagent Co., Ltd., China. Sodium borohydride was purchased from J&K Chemicals. Sodium persulfate (>98%) was from Shanghai Lingfeng Chemical Reagent Co. Ltd. O-NCB (>99%, GC grade), p-NCB (>99.5%, GC grade) and m-NCB (>98%, chemical pure) were all provided by Aladdin (Shanghai) Reagent Co. Ltd. Absolute ethanol and hexane were both chromatographically pure and obtained from Merk. The experimental ultrapure water was provided by Millipore pure water system. Other reagents are of analytical grade.

### 2.2 Preparation of nZVI/BC Composite

The preparation of peanut shell-derived biochar followed the procedure that was mentioned before (Wang et al., 2019). In brief, peanut shell was cleaned by water, dried (70°C, 24 h) and broken into about 2 mm strips, then pyrolyzed in a muffle at 500°C under limited oxygen condition for 2 h, then ground and passed through a 0.25 mm sieve. The nZVI/BC composite was then prepared using the liquid phase reduction method. To begin with, 5 g of ferrous sulfate heptahydrate was weighted and dissolved in 250 ml of 30% ethanol solution (volume fraction) in a three-necked flask. A certain amount of biochar (2 g) was added to the three-necked flask and mixed with ferrous sulfate heptahydrate solution, and nitrogen was then purged into the mixed solution to exclude the oxygen during the whole preparation procedure. Following, 100 ml of sodium borohydride solution at 0.395 mol L^−1^ was added dropwise through a constant pressure funnel, maintained at 1–2 drop s^−1^. With strong stirring by an electric stirring device, nZVI deposited on the surface of BC. After the completion of sodium borohydride addition, the solution was further stirred for 30 min with the nitrogen purging. The obtained nZVI/BC particles were settled and separated from the liquid by a positive pressure filter under an atmosphere of nitrogen, then washed with deionized water and anhydrous ethanol at least three times, then transferred to a screw-top reagent bottle, and dried in the oven at 70°C under protection of nitrogen, and stored under anaerobic conditions for use.

### 2.3 Batch Treatment Experiments

Batch aqueous tests were performed in 40 ml stoppered glass tubes with Teflon-lined screw caps. To begin with, 15 ml of NCB water solution (with initial concentration of each NCB was 5, 10, or 20 mg L^−1^) and 15 ml of sodium persulfate (with the amount of 3, 6, or 12 mmol) was added sequentially to the tubes and mixed with certain amount nZVI/BC particles (with Fe amount of 3 or 6 mmol). The tubes were placed in a rotary shaker (25 ± 1°C, 150 rpm). At regular time intervals, three tubes were sampled and sacrificed for analysis. The mixture was centrifugated at 3,000 rpm for 5 min to separate the solution from solid particles. Then 10 ml of solution phase was transferred and extracted with 10 ml of hexane. The composite particles were mixed with 2 ml ethanol and 8 ml hexane and extracted in a rotary shaker for 2 h, centrifugated and filtered. The organic phase was subjected for analysis. Triplicate samples were prepared for each test.

### 2.4 Analysis Method

The organic extractant was subjected for OCPs analysis on an Agilent 6890N gas chromatograph analyzer equipped with an electron capture detector and a HP-5 capillary column (30.0 mm × 0.32 mm × 0.25 µm). The injector and detector temperatures were 280 and 300°C, respectively, and the injection volume was 1.0 µl. The carrier gas flow rate (99.999% nitrogen) was 1.0 ml min^−1^. The heating procedure was as follows: the oven was heated to 90°C, held for 0.5 min and then heated from 90 to 110°C at a rate of 10°C min^−1^, and from 110 to 150 at a rate of 10°C min^−1^. Finally, the oven was heated to 270°C at a rate of 20°C min^−1^ and then held for 1 min.

### 2.5 Statistical Analysis

All data come from the average value of three repeated samples, statistically analyzed by SPSS 13.0 software. Data were analyzed by two way analysis of variance. The differences identified were pinpointed by an unpaired Student’s t-test. Mean values were compared by least significant difference at probability level≤5%. All charts were made by Microsoft Excel 2013 and Origin 2018 software.

## 3 Results and Discussion

### 3.1 Characteristics of BC and nZVI/BC

The composition of BC was analyzed on a Vario EL III Elemental Analyzer (German elementary company). The contents of C, H, O, and N were 61.3%, 2.69, 12.0, and 1.66%, respectively. The specific surface area of nZVI and nZVI/BC obtained from ASAP 2010N specific surface area analyzer were 34.2 and 6.3 m^2^ g^-1^, respectively.

**FIGURE 1:**
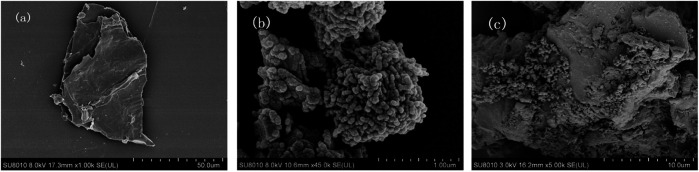
SEM images of BC **(A)**, nZVI **(B)**, nZVI/BC **(C)**.

Scanning electron microscope (SEM) was performed to examine the morphologies of BC, nZVI, and nZVI/BC on a JSM-7600F scanning electron microscope at an accelerating voltage of 10 kV. It can be observed from the SEM characterization image (Figure 1A) that the peanut-derived BC was of laminated structure with rough and porous surface morphologies. The nZVI was composed of fine spherical particles, and was severely agglomerated (Figure 1B). For the nZVI/BC composites, the nZVI particles were homogeneously distributed across the entire BC surface, forming a chain-like structure (Figure 1C). In addition, the diameters of spherical nZVI particles were about 30–40 nm.

### 3.2 NCB Degradation by Varied Materials

#### 3.2.1 PS Activation by Different nZVI/BC Materials

**FIGURE 2:**
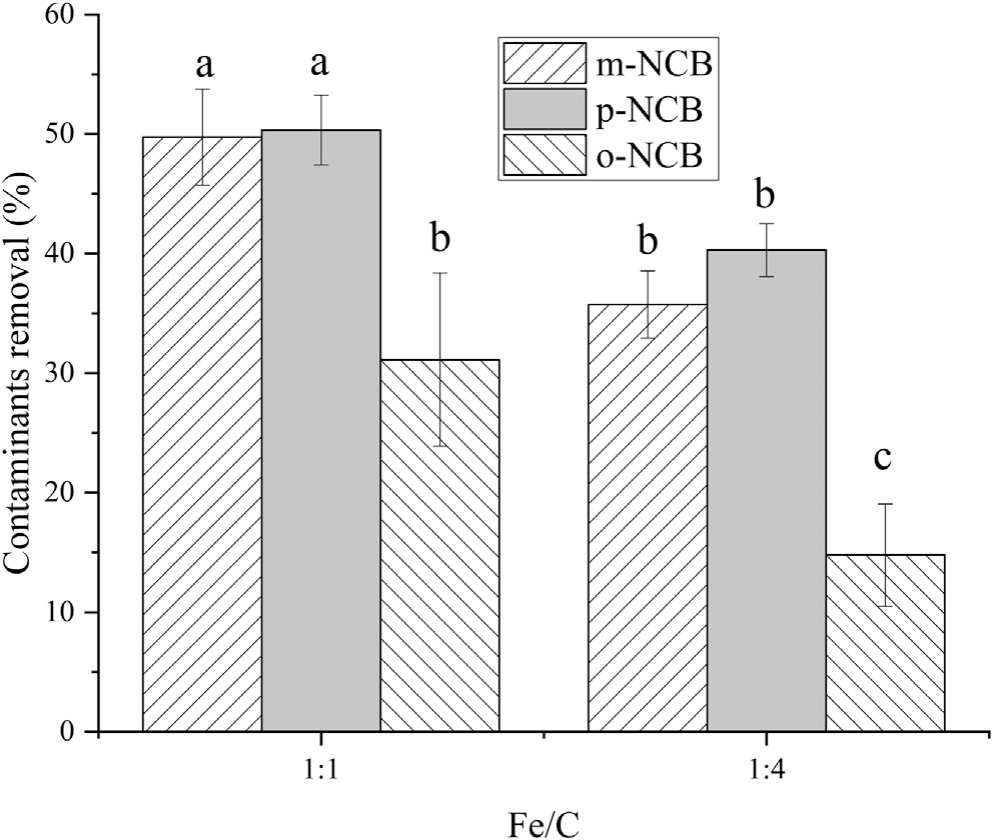
Effect of Fe/C in composite on NCB degradation. The nZVI/BC amount 0.01 g, Fe/PS molar ratio 1:1, the initial NCB isomer concentration 10 mg L^−1^, and the reaction time 2 h.

The effect of nZVI/BC composition on the activation of PS was examined. Two different nZVI/BC particles, with mass ratio of nZVI to BC of 1:1 and 1:4, were tested. The amount of nZVI/BC was 0.01 g, and the reaction time was 2 h. As demonstrated by Figure 2, the system with a higher Fe/BC ratio of 1:1 had statistically higher removal rates for all three NCB isomers compared to that of 1:4. Yan et al. (2015) reported a desired nZVI to BC mass ratio of 1:5, wherein the degradation of TCE increased as the nZVI/BC ratio increasing from 1:1 to 1:5, and decreased when the ratio further increased to 1:7. In another study by Hussain et al. (2017), the optimal nZVI/BC ratio for activation PS was found as 1:3, the degradation of nonylphenol at this ratio was superior to 1:1, 1:2, and 1:4. The activation capacity of nZVI/BC composite was suggested to be associated with their surface area that provided more reactive sites of Fe to activate PS. However, excessive BC may block the reactive sites of the iron surface and cause the aggregation of BC sheets (Hussain et al., 2017). It could be inferred that the optimal composition of nZVI and BC relates with the different characteristics of both BC (peanut shell-derived or rice straw-derived) and target contaminants.

#### 3.2.2 Effect of nZVI/BC and PS Dosage

**FIGURE 3:**
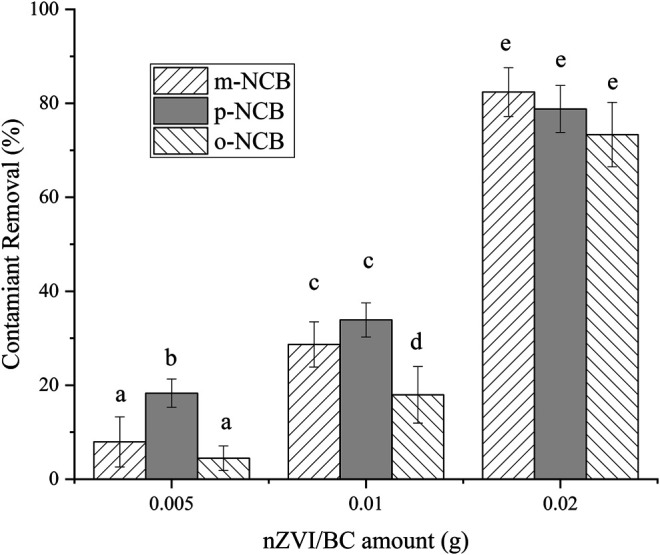
NCB degradation by different amount of nZVI/BC and PS. The Fe/C ratio 1:1, Fe/PS molar ratio 1:1, the initial NCB isomer concentration 10 mg L^−1^, and the reaction time 1 h.

The effect of nZVI/BC and PS amounts on NCB degradation was further investigated. 0.005–0.02 g of composite were used, and the Fe to PS molar ratio was kept as 1:1. As showed in Figure 3, no reliable removal of NCB was observed when 0.005 g of composite was added, especially for m- and o-NCB, wherein lower than 10% of removal were obtained. With the amount of nZVI/BC increasing to 0.01 and 0.02 g, the contaminants removal increased significantly. For instance, when 0.02 g of nZVI/BC (6 mmol L^−1^ of Fe) was added, the degradation rate of m-, p- and o-NCB reached 82.4%, 78.8%, and 73.3%, respectively. Higher activator and PS dosage meant more oxidation free radicals being formed and therefore, higher degradation efficiencies for contaminants (Luo et al., 2019). It is indicated that to achieve a desirable contaminant degradation rate, an amount of nZVI/BC of 0.02 g was required.

#### 3.2.3 NCB Degradation by Combined and Individual Systems

**FIGURE 4:**
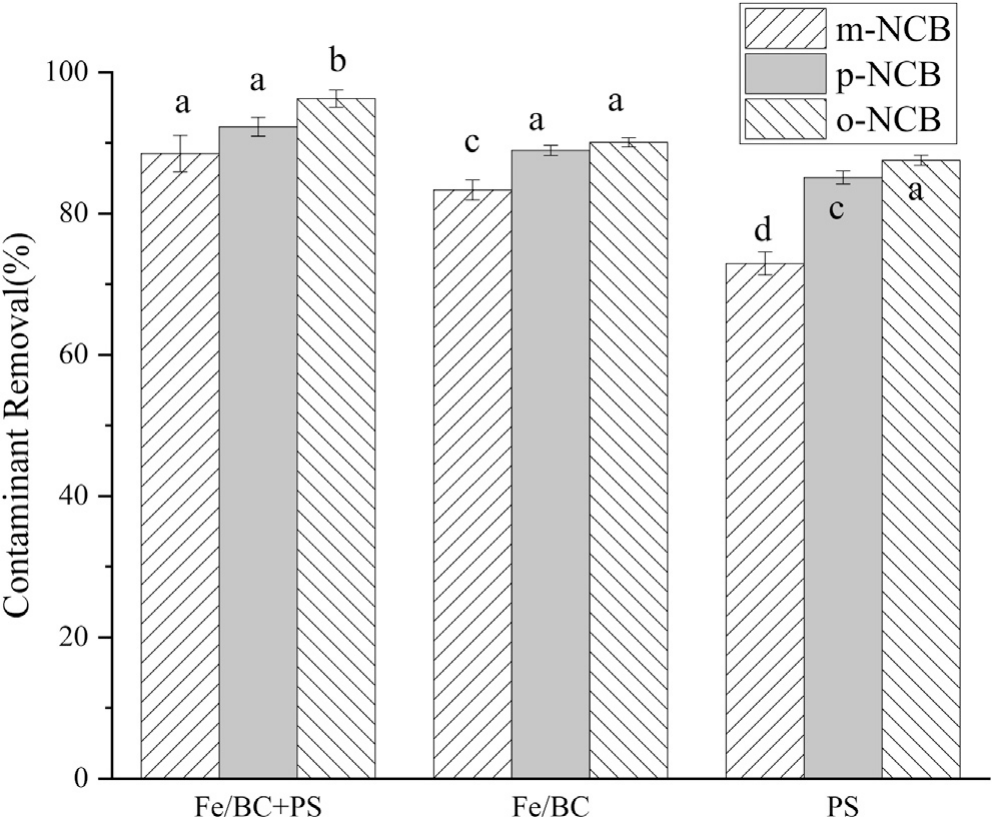
NCB degradation in different systems. The nZVI/BC amount 0.02 g, Fe/PS molar ratio 1:1, the initial NCB isomer concentration 10 mg L^−1^, and the reaction time 2 h.

Figure 4 reveals different removal performances of NCB in varied treatment systems, i.e., nZVI/BC, PS, and nZVI/BC activated PS. For all of the three treatments, robust contaminant removals were evidenced. It is suggested that the combination of nZVI/BC and PS exhibited superior removal efficacy to the system of PS alone. The promotive effect of nZVI/BC on PS oxidation has been verified in other studies (Yan et al., 2015; Luo et al., 2019). The promoted formation of SO_4_·-, which was detected as the primary active group, was supposed responsible for the accelerated degradation of NCB (Kang et al., 2019). Furthermore, both the Fe(II)/Fe(III) redox reaction induced by nZVI and the electron-transfer mediator of the BC oxygen functional groups were suggested to be related with promoting the generation of SO_4_·- in the combined nZVI/BC-PS system (Luo et al., 2019). Figure 2 suggests that no significant difference in NCB removal was obtained between nZVI/BC-PS and nZVI/BC anole system. Nevertheless, it should be noted that the oxidation reaction induced by nZVI/BC-PS could degrade the contaminants more thoroughly, which was comparatively superior to the reductive process by zero valent iron (Li et al., 2020). Herein, in the nZVI/BC-PS system, NCB could be reduced by nZVI to chloroaniline, and then oxidized by sulfate radicals to benzoquinone and finally been mineralized as carbon dioxide and water (Kang et al., 2019).

### 3.3 Effect of nZVI/BC Amount on NCB Degradation

**FIGURE 5:**
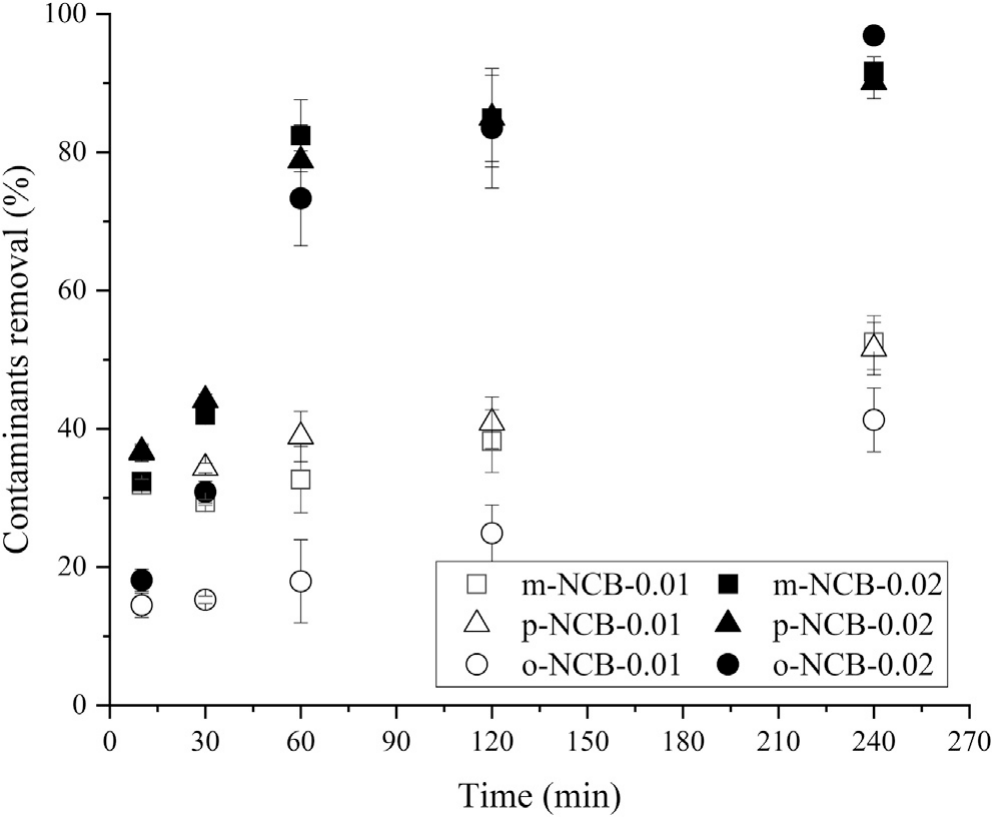
Effect of activator dosage on NCB degradation. The Fe/PS molar ratio 1:1, the initial NCB isomer concentration 10 mg L^−1^.

Figure 5 depicts the degradation kinetics of three NCB isomers by addition of different amount of PS activator (0.01 g vs. 0.02 g of nZVI/BC, corresponding to 3 and 6 mmol L^−1^ of Fe). It can be seen from Figure 5 that for all six groups tests, the degradation efficacies of NCB increased with the reaction time prolonging in the range of 0–240 min. Approximately 50% of m- and p-NCB were removed with 240 min when 0.01 g of nZVI/BC was used to activate PS. In comparison, significantly (*p* < 0.05) higher removal was obtained when nZVI/BC dosage was 0.02 g, wherein over 90% of NCB were degraded. The results were consistent with those in Section 3.2.2 and reported in other studies (Yan et al., 2015; Hussain et al., 2017). An increased dosage of nZVI/BC contributed to more active sites for PS decomposition, producing more SO_4_·- and increasing the efficiency of NCB degradation (Kang et al., 2019).

The much more rapid degradation of NCB at higher activator dosage can be also verified by the larger degradation first order kinetic constant (k_obs_), as showed in Table 1. In general, the k_obs_ at 0.02 g of Fe/BC were 5–11.75 times of those at 0.01 g, depending on the types of NCB isomers.

**TABLE 1:** Kinetics constants for aqueous OCPs degradation by varied ZVI systems.

Activator amount (G)	m-NCB		p-NCB		o-NCB	
	k _obs_ (/min)	*R* ^2^	k _obs_ (/min)	*R* ^2^	k_obs_ (/min)	*R* ^2^
0.01	0.0019 a	0.991	0.0014 a	0.973	0.0012 a	0.976
0.02	0.0095 b	0.939	0.0139 b	0.911	0.0141 b	0.977

Values with the same letter are not significantly different among treatments by LSD at the 5% level.

### 3.4 Effect of Fe:PS Ratio on NCB Degradation

The influence of Fe: PS molar ratio on NCB degradation was further investigated. The amount of nZVI/BC was kept as 0.02 g, i.e., the dosage of Fe was 6 mmol/L. Generally, for either p- or o-NCB, insignificant difference in contaminant degradation was attained among three Fe:PS ratio values (*p* > 0.05, Figure 5). However, the lower Fe/PS of 0.5 showed reduced degradation of m-NCB relative to Fe/PS of 2 and 1. For instance, the 4 h-removal of m-NCB for 1:1 Fe/PS group was 86.3%, while that for 1:2 Fe/PS group was 71.2%. It should be mentioned that herein the amount of Fe was constant, a lower Fe/PS value meant more or excess PS was initially present in the system, which might lead to the excessive scavenging of PS by itself, unfavorable to the oxidation of the contaminant (Hussain et al., 2017; Hayat et al., 2019). Yan et al. (2015) also reported a decreased TCE removal at lower nZVI/BC concentration beyond 4.5 mmol/L. The possible scavenging between SO_4_·- species themselves was proposed, as established in Eqs 4, 5. In another study, lower degradation of imidacloprid over higher nZVI dosage (above 3 g/L) or higher PS concentration (above 5 mmol/L) was evidenced, the self-scavenging caused by excess SO_4_·-−PS decomposition was investigated by detection of the concentration of PS anion during degradation experiments over applied concentrations of PS (Hayat et al., 2019).

(4) $$SO_{4}^{-}.+\;S_{2}O_{8}^{2-}\to SO_{4}^{2-}+S_{2}O_{8}^{-}$$

(5) $$SO_{4}^{-}.+\;SO_{4}^{-}\;\to S_{2}O_{8}^{2-}$$

### 3.5 Effect of NCB Initial Concentration

**FIGURE 6:**
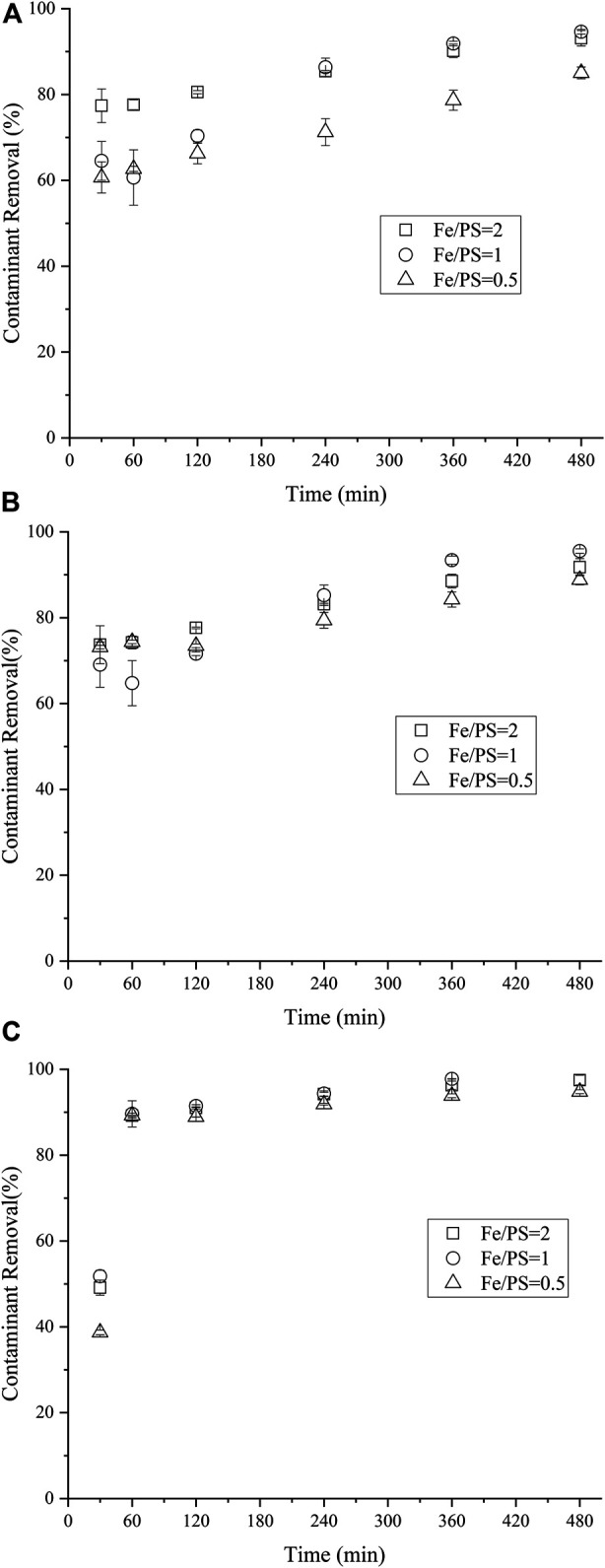
Effect of mole ratio of Fe/PS on m-NCB **(A)**, P-NCB **(B)** and o-NCB **(C)** degradation. The nZVI/BC amount 0.02 g, the initial NCB isomer concentration 10 mg L^−1^.

The influence of initial NCB concentration on their degradation was further investigated. The amount of nZVI/BC and PS was kept as 0.02 g and 6 mmol L^−1^, respectively. It can be seen from Figure 6 that the removal performance of NCB was significantly affected when the initial NCB concentration increased to 20 mg L^−1^ (*p* < 0.05). In particular, the removal rate for p-NCB was less than 30% within 480 min, while those were over 88% at initial concentrations of 5 and 10 mg L^−1^. Similar results have also been reported Liu et al. (2016), wherein insignificant concentration effect on the degradation of o-NCB at low o-NCB concentrations was found. As the formation of Fe^2+^ and SO_4_·- are initially rapid in the nZVI/PS system, the degradation over lower concentrations of NCB could be very fast. However, the concentration of racial became lower over higher NCB concentrations, thereby inducing a slower degradation rate.

**FIGURE 7:**
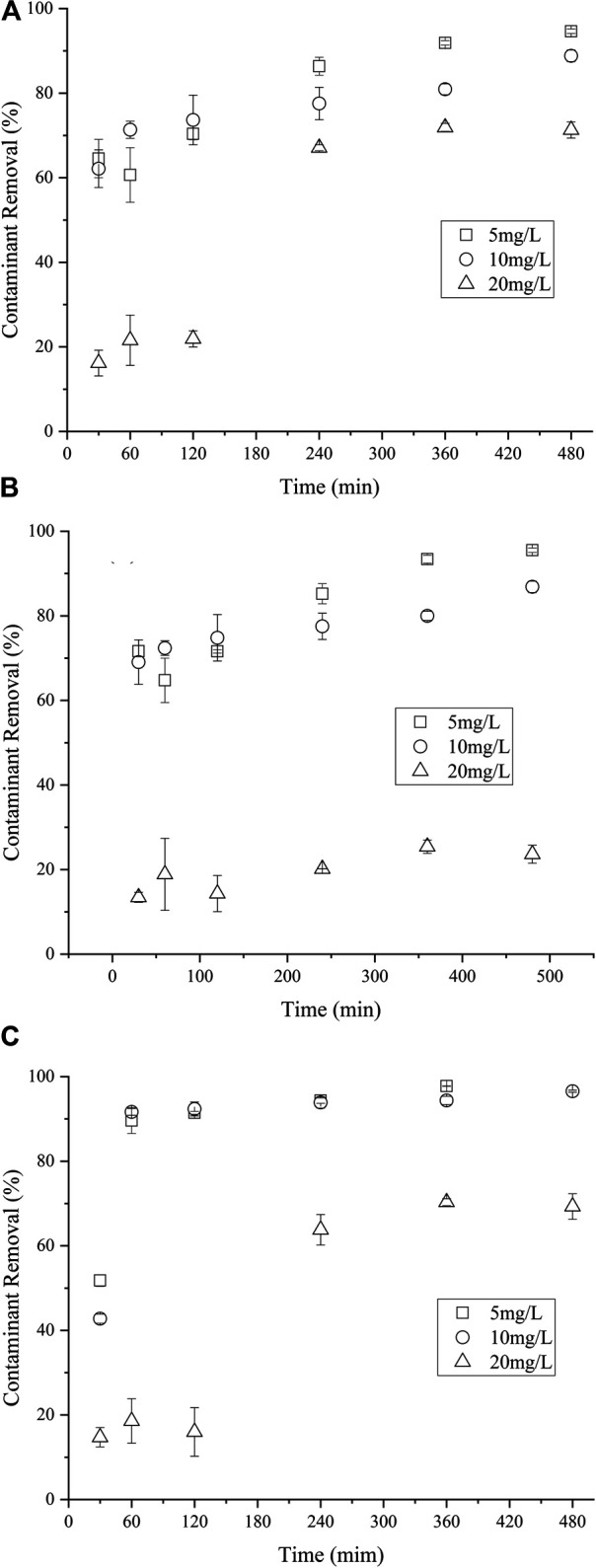
Effect of m-NCB **(A)**, P-NCB **(B)** and o-NCB **(C)** initial concentration on their degradation. The nZVI/BC amount 0.02 g, Fe/PS molar ratio 1:1.

## 4 Conclusion

In this study nZVI/BC was synthesized and used as an effective activator for PS oxidation to degrade NCB, a typical and toxic pesticide intermediate. The combination of composite and PS showed superior performance to PS alone. To some extent, a higher amount of nZVI/BC or PS exhibited larger NCB removal, while further increasing the dosage of the two agents led to decreasing oxidation effect, owing to the excessive scavenging of PS by itself. The optimal mole ratio of nZVI/BC to PS was found as 1:1. The initial concentration of the target contaminant had an insignificant influence in NCB removal, except for an initial concentration as high as 20 mg L^−1^. Our investigation suggests that nZVI/BC activated PS oxidation is an efficient method for NCB treatment in aqueous systems.

## Data Availability

The original contributions presented in the study are included in the article/Supplementary Material, further inquiries can be directed to the corresponding authors.
